# Preoperative Management of Catecholamine-Producing Pheochromocytomas and Paragangliomas—Results From a DELPHI Process

**DOI:** 10.1210/jendso/bvaf024

**Published:** 2025-02-14

**Authors:** Nicole Bechmann, Costanza Chiapponi, Harald-Thomas Groeben, Christian Grasshoff, Petra Zimmermann, Martin Walz, Martina Mogl, Volker Fendrich, Katharina Holzer, Nada Rayes, Matthias Kroiss

**Affiliations:** Institute of Clinical Chemistry and Laboratory Medicine, University Hospital Carl Gustav Carus, Medical Faculty Carl Gustav Carus, Technische Universität Dresden, Dresden 01307, Germany; Department of Surgery, Klinikum rechts der Isar, Technical University of Munich, School of Medicine, Munich 81675, Germany; Department of Anesthesiology, Critical Care Medicine and Pain Therapy, Kliniken Essen-Mitte, Essen 45136, Germany; Department of Anaesthesiology and Intensive Care Medicine, Tübingen University Hospital, Tübingen 72076, Germany; Department of General, Visceral and Transplant Surgery, University Hospital, LMU Munich, Munich 80336, Germany; Department of Surgery and Minimally Invasive Surgery, Kliniken Essen-Mitte, Essen 45136, Germany; Department of Surgery, Campus Charité Mitte, Campus Virchow-Klinikum, Charité—Universitätsmedizin Berlin, Corporate Member of Freie Universität Berlin, Humboldt-Universität zu Berlin, and Berlin Institute of Health, Berlin 10117, Germany; Department of Surgery, Schön Klinik Hamburg Eilbek, Hamburg 22081, Germany; Department of Visceral-, Thoracic- and Vascular Surgery, Philipps-University Marburg, Marburg 35043, Germany; Department of Visceral-, Transplant-, Thoracic and Vascular Surgery, University Hospital of Leipzig, Leipzig 04103, Germany; Department of Internal Medicine IV, University Hospital Munich, Munich 80336, Germany; Kroiss Endokrinologie und Diabetologie, Schweinfurt 97422, Germany

**Keywords:** pheochromocytoma, adrenal, hemodynamic instability, catecholamines, hypertensive crisis, phenoxybenzamine

## Abstract

**Context:**

European and German consensus guidelines advocate preoperative therapy with α-adrenoreceptor antagonists in symptomatic patients with catecholamine-producing pheochromocytomas and paragangliomas (PPGLs) to avoid hypertensive crisis during adrenalectomy. This practice has been questioned recently.

**Objective:**

This work aimed to assess current preoperative management of PPGLs across disciplines.

**Methods:**

The study was conducted from November 2023 to February 2024 using the Delphi technique. Two consecutive surveys were conceived by a steering group and 46 experts were consulted using REDCap web application (response: 74%).

**Results:**

There was general agreement about diagnostic tools and indication for adrenalectomy. In contrast, 20% of the panelists routinely administered α-adrenoreceptor antagonists to all patients, 50% only in case of symptoms, and about one-third of experts abandoned preoperative α-adrenoreceptor blockade. The prevention of anticipated intraoperative hypertensive crisis and cardiovascular complications (75%) as well as medicolegal considerations (25%) were the main motivations. Despite availability of short-acting α-adrenoreceptor antagonists, most experts (63%) continued to use phenoxybenzamine. Half of the experts preferred pretreatment in an outpatient setting, 13% routinely treated in the hospital, and 37% combined outpatient and inpatient treatment. Intraoperatively, urapidil and nitroprusside natrium were mainly used for blood pressure control. Postoperatively, around 60% of the experts routinely admitted patients to an intensive care or intermediate care unit.

**Conclusion:**

Current guideline recommendations for preoperative treatment with α-adrenoreceptor antagonists in patients with PPGLs are generally adopted by treating teams but current practice is very heterogeneous even among expert centers. With the improvement of surgical techniques and intraoperative management, a more individualized approach may be considered.

Paragangliomas are rare neuroendocrine tumors that arise from chromaffin cells of the adrenal medulla, also known as pheochromocytomas, or from the extra-adrenal sympathetic ganglia [1]. The continuous or paroxysmal release of epinephrine, norepinephrine, and/or dopamine from pheochromocytomas and paragangliomas (PPGLs) may cause permanent or paroxysmal arterial hypertension and more nonspecific signs and symptoms such as headache, palpitations, diaphoresis, and pallor. Cardiovascular sequelae are a potentially life-threatening complication of PPGLs [2]. Among adrenal incidentalomas, pheochromocytomas account for approximately 5% of diagnoses [3]. Among these patients, 20% to 50% might be normotensive and/or entirely asymptomatic [4-6]. In others, signs and symptoms of catecholamine excess are frequently overlooked [7]. Surgical tumor removal is the standard treatment for PPGLs [8]. For pheochromocytomas, the principal surgical approaches are by laparoscopic or retroperitoneoscopic adrenalectomy. Patients with hereditary disease such as multiple endocrine neoplasia type 2 (MEN2), von Hippel-Lindau disease, and neurofibromatosis type 1 have a higher risk of developing bilateral disease, which can be either synchronous or metachronous. In these patients, partial adrenalectomy (PA) might be considered to preserve adrenal function and prevent adrenal insufficiency [8], even though repeat surgery for secondary tumors may be necessary. Indeed, PA, especially in patients with hereditary PPGL, is associated with a higher risk of local recurrence [9], but the disease-specific mortality is comparable to that of radical adrenalectomy [10]. Therefore, PA appears to be safe when performed as a second operation in bilateral disease [10]. Intraoperative mobilization of the tumor often results in a sudden increase of blood pressure due to release of catecholamines. European and German guidelines therefore recommend treatment of symptomatic patients with α-adrenoreceptor antagonists preoperatively [8, 11]. However, this recommendation is based on weak evidence and there are at least 2 metanalyses questioning the benefit of preoperative α-adrenoreceptor blockade [12, 13]. Phenoxybenzamine, a noncompetitive α 1- and α 2-adrenoreceptors antagonist, has been commonly used to prepare patients for surgery. Prolonged hypotension after PPGL removal is, however, a dreaded complication and may require postoperative catecholamine administration in an intensive care unit [14]. During past years, more selective drugs like urapidil were proposed and successfully used in small case series [15, 16]. Recent studies have even shown that resection of pheochromocytomas without any preoperative α-adrenoreceptor blockade may be safe in experienced centers [17, 18].

Due to lack of evidence, as there are no randomized controlled trials, the preoperative management of PPGLs differs between endocrine centers. Therefore, the “Section for adrenal, steroids and hypertension” of the German Society of Endocrinology (DGE) and the “Surgical Working Group for Endocrinology” (CAEK) of the German Society for General and Visceral Surgery (DGAV) joined forces to collect data from an interdisciplinary board of experts at endocrine centers in Germany. The aim of this study was to reach a consensus on the optimal perioperative and intraoperative management to improve the postoperative outcome of surgery for PPGLs.

## Materials and Methods

The Delphi technique served as the basis for this consensus analysis. As part of this technique, consecutive surveys were sent to participants selected by the steering group (N.B., C.C., H.G., C.G., M.W., M.M., V.F., N.R., K.H., and M.K.) on the strength of their expertise in the management of catecholamine-producing PPGLs. The panel included 46 experts from different medical centers within Germany: 15 endocrine surgeons, 12 anesthesiologists, and 19 endocrinologists from university hospitals (n = 32), other public hospitals (n = 11), or private practices (n = 3). The study was performed from November 2023 to February 2024. The surveys were conducted online using REDCap, a secured web application [19, 20]. The Delphi process required 2 rounds of surveys to reach adequate consensus. In the first round, the questionnaire was mainly focused on the diagnosis of PPGLs and the factors influencing preoperative management. Based on the results of the first round, the second round included questions about the practical implementation of preoperative management. Responses were collected anonymously and monitored by 2 members of the steering group (N.B. and M.K.). In both rounds a response rate of 74% was reached (n = 34), including 12 surgeons, 12 or 13 endocrinologists, and 9 or 10 anesthesiologists. All answers were afterward intensively discussed by the steering group.

## Results

### Diagnosis and Indication for Surgery

**Figure 1.**
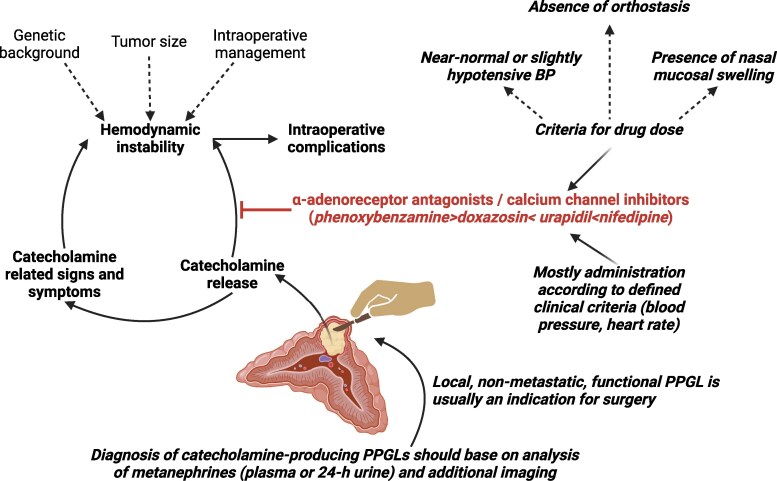
Perioperative workflow for catecholamine-producing pheochromocytomas and paragangliomas (PPGLs). The statements highlighted in italics indicate the consensus or approach of the expert panel. Created with BioRender.

The experts agreed that the diagnosis of catecholamine-producing PPGLs should be based on the analysis of metanephrines in plasma or 24-hour urine and the application of an imaging technique (94% consent; Fig. 1). Conventional cross-sectional imaging should be complemented by functional imaging (MIBG scintigraphy, DOPA-PET-CT, DOTATOC/DOTATATE-PET-CT) in patients with PPGLs larger than 4 cm (29%), aged 45 years or older (32%), extra-adrenal location (77%), or multifocal tumors (74%). The diagnosis of a local, nonmetastatic, functional PPGL is usually an indication for surgery (88% consent). Most experts aimed for a period of 2 to 4 weeks between diagnosis and surgery (53%) or less than 2 weeks (24%).

While experts agreed that disease-related signs and symptoms should be assessed systematically (97% agreement), only 17.6% considered clinical symptoms to be a suitable marker for the guidance of preoperative management (Table 1). The majority of experts (97%) believed that the early and systematic recording of cardiovascular comorbidities is necessary. For this purpose, 75% carried out blood pressure measurements in the practice/outpatient clinic, 66% performed 24-hour blood pressure measurements, 41% occasional measurements of blood pressure and pulse by the patient, 41% transthoracic echocardiograms, and 16% 24-hour echocardiography. An argument against routine transthoracic echocardiography was its inability to predict cardiac arrhythmias, which are supposed to be the main intraoperative risk. Therefore, it was not considered to be useful for risk assessment in general.

**Table 1.** Current approaches in the preoperative management of catecholamine-producing pheochromocytomas and paragangliomas in Germany

	Experts’ approach	Consensus or standardized approach
Pretreatment with α-adrenoreceptor antagonists or calcium channel inhibitors	52% always pretreat 21% only in certain cases 27% never pretreat	No
When is pretreatment required?	Only 9% are convinced that pretreatment is always required, but 53% think pretreatment is required in symptomatic patients	No
Reasons for α-adrenoreceptor blockade	67% prevention of blood pressure crises 71% cardiovascular complications 54% reduction of symptoms 25% fear of legal consequences (neglecting guidelines) 17% tradition	NA
Does the ability to provoke clinical symptoms change preoperative management?	No (80%)	Yes
Which drug is used for pretreatment?	88% phenoxybenzamine 42% doxazosin 33% urapidil 4% nifedipine	No
Why is phenoxybenzamine still used?	63% vast experience with the drug and its long-lasting antihypertensive effect 33% use alternative drugs because of the long postoperative overhang and prolonged hypotension of phenoxybenzamine.	NA
Main criterion for guidance of drug dose	67% near-normal or slightly hypotensive blood pressure 21% absence of orthostasis 17% presence of nasal mucosal swelling	No
Period of pretreatment	2-3 days (urapidil) and 2 weeks (phenoxybenzamine)	NA (depends on drug)
Initiation or adjustment of antihypertensive medication	6% always change of antihypertensive medication 27% make changes only in patients with documented blood pressure values >160 mm Hg systolic or >95 mm Hg diastolic 50% only in patients with documented blood pressure values >140 mm Hg systolic or >90 mm Hg diastolic 12% only in symptomatic patients 6% never change antihypertensive medication	No

Abbreviation: NA, not applicable.

There was disagreement about the need for systematic testing for MEN2 or other predisposing syndromes prior to surgery: The arguments were that first, genetic analysis needs time and the operation should not be delayed, and, second that the consequences for the preoperative and perioperative management of PPGL patients remain unclear. Nevertheless, genetic testing was recommended within the first 4 weeks after diagnosis. As a more practical screening tool, a relative majority of experts (49%) was in favor of including calcitonin testing in the preoperative screening of PPGLs patients to reduce the clinical likelihood of MEN2. Only 27% were against calcitonin testing or considered it useful only for epinephrine-producing PPGLs.

### Preoperative Management of Patients With Pheochromocytomas and Paragangliomas

Most experts on the panel still pretreated their PPGL patients with α-adrenoreceptor antagonists or calcium channel inhibitors (52%), some only in a subset of cases (21%), and 27% had abandoned pretreatment. However, when asked whether an α-adrenoreceptor antagonist or a calcium channel inhibitor treatment is generally required, only 9% answered yes. When the question was specified to symptomatic PPGL patients, 53% of experts considered preoperative treatment to be necessary. Despite the fact that only a minority of experts were convinced that preoperative α-adrenoreceptor receptor blockade reliably prevents intraoperative hypertensive crises (systolic blood pressure increase > 200 mm Hg, 18%) and reduces the perioperative risk of cardiovascular or cerebral complications (33%), a large proportion preferred pretreatment. The main reasons for α-adrenoreceptor blockade, apart from the presumed prevention of blood pressure crises (67%, multiple answers possible) and cardiovascular complications (71%) and the reduction of symptoms (54%), were the fear of legal consequences (neglecting of guidelines, 25%) and tradition (“we have always done it this way,” 17%). Of the experts who stated that they would perform preoperative treatment, one-third would administer treatment even in asymptomatic patients without preexisting cardiac conditions. A further 33% pretreated only symptomatic PPGL patients, 13% pretreated patients with a cardiac risk, and 8% decided individually according to other criteria. These criteria included the presence of preoperative symptoms and history of hypertensive crisis. If the PPGL patient reports being able to provoke clinical symptoms, most of the experts would not change the preoperative management of the patient (80%), the others would optimize the α-adrenoreceptor blockade or adjust the dose under these circumstances.

Phenoxybenzamine was frequently used for pretreatment (88%; multiple selections possible), followed by doxazosin (42%), urapidil (33%), and nifedipine (4%). Most of the experts (63%) preferred phenoxybenzamine because of their ample personal experience with the drug and its long-lasting antihypertensive effect, whereas 33% used alternative drugs due to the fear of prolonged postoperative hypotension. Only 38% of the experts always chose the same medication for surgical pretreatment, while 47% used different medications depending on patient's symptoms or whether inpatient or outpatient medication was required. In most centers (65%), an α-adrenoreceptor antagonist or a calcium channel inhibitor was administered according to defined clinical criteria (blood pressure, heart rate) and not according to a fixed schedule (9%). In the preoperative setting, antihypertensives were usually administered orally (90%) and only rarely intravenously (10%). The main criterion for dosing of the drug was to aim for near-normal or slightly hypotensive blood pressure (67%) in addition to the absence of orthostasis (21%) and the presence of nasal mucosal swelling (17%). Tumor size had no influence on the administered dose of α-adrenoreceptor antagonist or calcium channel inhibitor.

In most centers, preoperative treatment was carried out on an outpatient basis (50%) and in only 13% of the centers on an inpatient basis, while 38% either started on an outpatient basis and then switched to inpatient treatment, depending on the severity of the symptoms of the antihypertensive drugs. The period of pretreatment varied between 2 and 3 days (urapidil) and 2 weeks (phenoxybenzamine).

Regarding preexisting antihypertensive medication, 97% of the experts believed that the medication should be continued if blood pressure is well controlled. Initiation or adjustment of antihypertensive medication was always carried out by 6% of experts, while 27% made changes only in patients with documented blood pressure values greater than 160 mm Hg systolic or greater than 95 mm Hg diastolic, 50% only in patients with documented blood pressure values greater than 140 mm Hg systolic or greater than 90 mm Hg diastolic, 12% only in symptomatic patients, and 6% of experts never change antihypertensive medication.

### Complications During Preoperative Management

Complications during preoperative pretreatment with α-adrenoreceptor antagonist or a calcium channel inhibitor had already been encountered by 52% of experts in the panel in their daily clinical routine. Most of the complications occurred in connection with the administration of phenoxybenzamine (69% vs 26% with doxazosin, 5% with urapidil). Complications included hypotension and orthostatic syncope. Persistent hypotension postoperatively and reduced response to catecholamines intraoperatively and postoperatively were also described.

### Surgery and Intraoperative Management of Pheochromocytomas and Paragangliomas

All experts agreed that PPGLs should only be operated at experienced centers by endocrine surgeons. Most of the experts (88%) believed that the intraoperative management of PGGLs differs from that of other vascular or visceral surgical procedures because, for example, fluctuations in blood pressure have to be compensated for with short-term medication.

More than 5 PPGL operations per year were considered to be required for an experienced center by most of the experts. In this context, it was discussed that operative experience can also result from adrenalectomies in other hormone-active and hormone-inactive tumors and should be considered accordingly.

Even though most experts (88%) agreed that manifest cardiovascular comorbidity should potentially change intraoperative management, it became clear that there are no standardized recommendations. The majority advised the insertion of a central venous catheter for circulatory control (68%) and the application of arterial blood pressure measurement (88%). The experts considered hypotension (74%) to be more critical than hypertension (26%).

However, most experts were unable to define a local maximum tolerated intraoperative systolic blood pressure (68%). The upper limit for those who have standards defined was between 140 and 200 mm Hg. Urapidil or sodium nitroprusside were usually used to treat intraoperative hypertension, less frequently esmolol. When asked which factors influence the intraoperative management of hypertension, 64% stated the dynamics of the increase in blood pressure and comorbidities. The patient's age and sex were also considered (39%). Less frequently, type of hormone overproduction (epinephrine or norepinephrine) and heart rate were stated as additional factors. Intraoperative hypotension was mostly treated with a combination of volume increase and norepinephrine or vasopressin (65%), less frequently with norepinephrine (35%), vasopressin (15%), or volume administration (32%) alone. In most centers, patients were routinely admitted to an intensive care unit or intermediate care unit (61%) for at least 1 day after adrenalectomy.

## Discussion

Surgical procedures for PPGLs may cause intraoperative and postoperative hemodynamic (HD) changes, so-called instability, due to the catecholamine release of these rare tumors. Although a consensus was reached among the interdisciplinary expert panel on diagnostic tools and indication for adrenalectomy in catecholamine-producing PPGLs, implementation of preoperative α-adrenoreceptor blockade differed fundamentally in terms of (a) whether and according to which criteria α-adrenoreceptor blockade is performed, (b) which drug is administered and at what dose, and (c) the criteria according to which α-adrenoreceptor blockade is performed (see Table 1 and Fig. 1). Current guidelines for preoperative treatment with α-adrenoreceptor antagonists in patients with PPGLs are based on studies with nonspecific, long-acting drugs; therefore, multidisciplinary, prospective multicenter studies are needed to provide a thorough evaluation to improve patient management.

Although the experts agreed that the diagnosis of local, nonmetastatic, catecholamine-producing PPGL by metanephrines analysis (plasma or 24-hour urine) and additional imaging is a clear indication for surgery, only half of the experts still use α-adrenoreceptor blockade in all patients, despite existing guidelines [8, 11].

The DELPHI process demonstrated that there is great uncertainty about which and whether any patients benefit from pretreatment and for whom it might be indispensable. Two studies initiated in Germany provide evidence that pheochromocytoma resection is safe without α-adrenoreceptor blockade pretreatment in experienced centers [17, 18], with even lower rates of cardiovascular complications and mortality in nonpretreated patients compared to patients pretreated with α-adrenoreceptor blockade [17]. Even at high doses of α-adrenoreceptor–blocking agents, systolic blood pressure greater than 200 mm Hg has been observed, while at the same time the incidence of intraoperative hypotension increased significantly in patients receiving α-adrenergic–blocking agents [18], exposing patients to additional potential risks. These studies challenged the routine use of α-adrenoreceptor blockers and encouraged other centers in Germany to question their administration, which is also reflected in the study presented. Thus, 27% of the experts stated that they no longer pretreat their PPGL patients with α-adrenoreceptor antagonists or calcium channel inhibitors.

In addition, there are many variations in the implementation of α-adrenoreceptor blockade, for example, in terms of medication and administration. Therefore, factors that allow the assessment of intraoperative and perioperative risks of each individual patient are needed. A retrospective, single-center study identified high plasma norepinephrine concentrations, larger tumor size, greater drop in postural blood pressure after α-adrenoreceptor blockade, and mean arterial pressure greater than 100 mm Hg (130/85 mm Hg) as potential risk factors for HD instability during pheochromocytoma surgery, with comparable efficacy in preventing HD instability with phenoxybenzamine and doxazosin [21]. Data from France presented at the American Association of Endocrine Surgeons showed there is a significant variability in HD instability between centers and surgeons, suggesting that surgical aspects of preparation and clinical experience also play an important role [22]. Another study found that, in addition to tumor size, open adrenalectomy and type of α-adrenoreceptor blockade correlated with intraoperative HD instability during pheochromocytoma resection [23]. Retroperitoneal adrenalectomy carries a slightly higher risk of mean arterial pressure less than 60 mm Hg compared to transperitoneal adrenalectomy, but overall cardiovascular morbidity rates are comparable between the two procedures [24]. Interestingly, the same study also showed that the variability in the institutional intraoperative management of pheochromocytomas has a significant effect on HD instability, which underlines the importance of standardization. Uncertainty about potential patient selection criteria is reflected in the highly heterogeneous clinical practice even among expert centers.

The symptomatic treatment of catecholamine excess especially in patients with heart failure or cardiac arrhythmias could not be covered in this DELPHI process due to the multiplicity of factors that may influence treatment decisions. These patients require individualized therapy and close interaction between the patient and the treating physician.

There is disagreement among experts about the timing of genetic testing (often not feasible before surgery) and the resulting implications for preoperative management. As a more practical screening tool, the majority (48.5%) were in favor of including calcitonin testing in the preoperative screening of PPGLs patients to rule out the presence of possible MEN2. Only 27.3% were against it or considered it useful only for epinephrine-producing PPGLs. However, MEN2 patients with pheochromocytoma have smaller tumors but do not differ from non-MEN2 patients in terms of hypertensive episodes during surgery [25]. A Chinese cohort showed similarly to previous studies an enrichment of PPGL patients with somatic pathogenic variants in *HRAS* compared to European cohorts [26, 27]. Genetic background, tumor size, age, and the presence of catecholamine-related signs and symptoms were associated with HD instability in a Chinese cohort [28].

The DELPHI technique used in this study has certain limitations. It reflects only the opinion of selected experts in a specific field. In the present study, experts from different disciplines (surgeons, endocrinologists, and anesthesiologists) were included because the complex preoperative management of patients with catecholamine-producing PPGLs requires the close interaction of all 3 disciplines. Nevertheless, not all experts are equally involved in all preoperative and intraoperative decisions, which can lead to a bias in the responses. To minimize this risk, especially in the second round of the survey, we allowed respondents to give a “don’t know” answer. For rare diseases such as PPGLs, however, experts are needed to identify deficits in patient care. Furthermore, the surveys reflect current practice in Germany, which may be biased by clinical availability and reimbursement in this particular setting. This could be considered a shortcoming and limit the applicability of some conclusions to other health-care systems. For example, the decision to perform functional imaging may be particularly susceptible to such bias. On the other hand, despite the relatively well-defined setting of our survey, there were controversial issues that may have prevented consensus if conducted at an international level.

Prospective multicenter studies are needed to finally clarify whether and to what extent preoperative pretreatment of catecholamine-producing PPGLs is necessary and, most important, for which patients. Criteria that reliably identify individual patients at increased risk need to be identified and further validated. In the absence of such evidence, an individualized medical approach is required that includes taking a careful clinical history and thorough assessment of factors that potentially may lead to adverse clinical outcomes, but that also considers the local expertise and the availability of diagnostic and therapeutic procedures. Alongside cardiovascular criteria, the presence of cystic or necrotic lesions has been proposed. An in vitro study suggests that necrotic changes may lead to an accumulation of free catecholamines [29].

The prevailing heterogeneity in the implementation and applied criteria for preoperative management of PPGLs calls for more caution in interdisciplinary guidelines. This is particularly true for the availability of advanced preoperative and intraoperative treatment options. Due to the complexity of the perioperative management, surgery for PPGLs requires an experienced multidisciplinary team of—at least—endocrinologists, surgeons, and anesthesiologists. More important, the exchange between disciplines and a careful assessment of outcomes and the definition of benchmarks is necessary to improve patient safety.

## Data Availability

Some or all data sets generated during and/or analyzed during the present study are not publicly available but are available from the corresponding author on reasonable request.

## Abbreviations

HD: hemodynamic
MEN2: multiple endocrine neoplasia type 2
PA: partial adrenalectomy
PPGLs: pheochromocytomas and paragangliomas

## References

[1] Mete O, Asa SL, Gill AJ, Kimura N, de Krijger RR, Tischler A. Overview of the 2022 WHO classification of paragangliomas and pheochromocytomas. Endocr Pathol. 2022;33(1):90‐114. 35285002 10.1007/s12022-022-09704-6

[2] Prejbisz A, Lenders JW, Eisenhofer G, Januszewicz A. Cardiovascular manifestations of phaeochromocytoma. J Hypertens. 2011;29(11):2049‐2060. 21826022 10.1097/HJH.0b013e32834a4ce9

[3] Fassnacht M, Tsagarakis S, Terzolo M, et al European Society of Endocrinology clinical practice guidelines on the management of adrenal incidentalomas, in collaboration with the European Network for the study of adrenal tumors. Eur J Endocrinol. 2023;189(1):G1‐G42. 37318239 10.1093/ejendo/lvad066

[4] Mantero F, Terzolo M, Arnaldi G, et al A survey on adrenal incidentaloma in Italy. J Clin Endocrinol Metab. 2000;85(2):637‐644. 10690869 10.1210/jcem.85.2.6372

[5] Kopetschke R, Slisko M, Kilisli A, et al Frequent incidental discovery of phaeochromocytoma: data from a German cohort of 201 phaeochromocytoma. Eur J Endocrinol. 2009;161(2):355‐361. 19497985 10.1530/EJE-09-0384

[6] Grozinsky-Glasberg S, Szalat A, Benbassat C, et al Clinically silent chromaffin-cell tumors: tumor characteristics and long-term prognosis in patients with incidentally discovered pheochromocytomas. J Endocrinol Invest. 2010;33(10):739‐744. 20479567 10.1007/BF03346680

[7] Rogowski-Lehmann N, Geroula A, Prejbisz A, et al Missed clinical clues in patients with pheochromocytoma/paraganglioma discovered by imaging. Endocr Connect. 2018;7(11):1168‐1177. 30352425 10.1530/EC-18-0318 PMC6215794

[8] Lenders JW, Duh Q-Y, Eisenhofer G, et al Pheochromocytoma and paraganglioma: an endocrine society clinical practice guideline. J Clin Endocrinol Metab. 2014;99(6):1915‐1942. 24893135 10.1210/jc.2014-1498

[9] Zawadzka K, Tylec P, Małczak P, Major P, Pędziwiatr M, Pisarska-Adamczyk M. Total versus partial adrenalectomy in bilateral pheochromocytoma–a systematic review and meta-analysis. Front Endocrinol (Lausanne). 2023;14:1127676. 36998480 10.3389/fendo.2023.1127676 PMC10043479

[10] Xu K, Langenhuijsen JF, Viëtor CL, et al PRAP study—partial versus radical adrenalectomy in hereditary pheochromocytomas. Eur J Endocrinol. 2024;191(3):345‐353. 39171965 10.1093/ejendo/lvae108

[11] Lorenz K, Langer P, Niederle B, et al Surgical therapy of adrenal tumors: guidelines from the German association of endocrine surgeons (CAEK). Langenbecks Arch Surg. 2019;404(4):385‐401. 30937523 10.1007/s00423-019-01768-z

[12] Schimmack S, Kaiser J, Probst P, Kalkum E, Diener MK, Strobel O. Meta-analysis of α-blockade versus no blockade before adrenalectomy for phaeochromocytoma. Journal of British Surgery. 2020;107(2):e102‐e108. 10.1002/bjs.11348 31903584

[13] Wang J, Liu Q, Jiang S, et al Preoperative α-blockade versus no blockade for pheochromocytoma–paraganglioma patients undergoing surgery: a systematic review and updated meta-analysis. Int J Surg. 2023;109(5):1470‐1480. 37037514 10.1097/JS9.0000000000000390 PMC10389437

[14] Van der Zee PA, De Boer A. Pheochromocytoma: a review on preoperative treatment with phenoxybenzamine or doxazosin. Neth J Med. 2014;72(4):190‐201. 24829175

[15] Habbe N, Ruger F, Bojunga J, Bechstein WO, Holzer K. Urapidil in the preoperative treatment of pheochromocytomas: a safe and cost-effective method. World J Surg. 2013;37(5):1141‐1146. 23381676 10.1007/s00268-013-1933-9

[16] Tauzin-Fin P, Barrucand K, Sesay M, et al Peri-operative management of pheochromocytoma with intravenous urapidil to prevent hemodynamic instability: a 17-year experience. J Anaesthesiol Clin Pharmacol. 2020;36(1):49‐54. 32174657 10.4103/joacp.JOACP_71_18 PMC7047675

[17] Groeben H, Walz M, Nottebaum B, et al International multicentre review of perioperative management and outcome for catecholamine-producing tumours. Br J Surg. 2020;107(2):e170‐e178. 31903598 10.1002/bjs.11378 PMC8046358

[18] Groeben H, Nottebaum B, Alesina P, Traut A, Neumann H, Walz M. Perioperative α-receptor blockade in phaeochromocytoma surgery: an observational case series. Br J Anaesth. 2017;118(2):182‐189. 28100521 10.1093/bja/aew392

[19] Harris PA, Taylor R, Thielke R, Payne J, Gonzalez N, Conde JG. Research electronic data capture (REDCap)—a metadata-driven methodology and workflow process for providing translational research informatics support. J Biomed Inform. 2009;42(2):377‐381. 18929686 10.1016/j.jbi.2008.08.010 PMC2700030

[20] Harris PA, Taylor R, Minor BL, et al The REDCap consortium: building an international community of software platform partners. J Biomed Inform. 2019;95:103208. 31078660 10.1016/j.jbi.2019.103208 PMC7254481

[21] Bruynzeel H, Feelders R, Groenland T, et al Risk factors for hemodynamic instability during surgery for pheochromocytoma. J Clin Endocrinol Metab. 2010;95(2):678‐685. 19965926 10.1210/jc.2009-1051

[22] Nomine-Criqui C, Delens A, Nguyen-Thi P-L, et al Intraoperative Hemodynamic Instability During Laparoscopic Adrenalectomy for Pheochromocytoma Without Preoperative Medical Preparation Versus Non-Secreting Tumor. Abstract American Association of Endocrine Surgeons; 2024.

[23] Kiernan CM, Du L, Chen X, et al Predictors of hemodynamic instability during surgery for pheochromocytoma. Ann Surg Oncol. 2014;21(12):3865‐3871. 24939623 10.1245/s10434-014-3847-7 PMC4192065

[24] Vorselaars WM, Postma EL, Mirallie E, et al Hemodynamic instability during surgery for pheochromocytoma: comparing the transperitoneal and retroperitoneal approach in a multicenter analysis of 341 patients. Surgery. 2018;163(1):176‐182. 29122324 10.1016/j.surg.2017.05.029

[25] Scholten A, Vriens MR, Cromheecke GJE, Borel Rinkes IH, Valk GD. Hemodynamic instability during resection of pheochromocytoma in MEN versus non-MEN patients. Eur J Endocrinol. 2011;165(1):91‐96. 21498631 10.1530/EJE-11-0148

[26] Jiang J, Zhang J, Pang Y, et al Sino-European differences in the genetic landscape and clinical presentation of pheochromocytoma and paraganglioma. J Clin Endocrinol Metab. 2020;105(10):3295‐3307. 10.1210/clinem/dgaa502 32750708

[27] Richter S, Bechmann N. Patient sex and origin influence distribution of driver genes and clinical presentation of paraganglioma. J Endocr Soc. 2024;8(5):bvae038. 38481600 10.1210/jendso/bvae038 PMC10928507

[28] Li M, Zhang J, Pang Y, et al Genetic background and intraoperative haemodynamic instability in patients with pheochromocytoma and paraganglioma: a multicenter retrospective study. Int J Surg. 2024;111(1):913‐919. 10.1097/JS9.0000000000001995 PMC11745656 39093877

[29] Bechmann N, Poser I, Seifert V, et al Impact of extrinsic and intrinsic hypoxia on catecholamine biosynthesis in absence or presence of HIF2α in pheochromocytoma cells. Cancers (Basel). 2019;11(5):594. 31035382 10.3390/cancers11050594 PMC6562431

